# Supplementary figures and images for: Recent mobility of plastid encoded group II introns and twintrons in five strains of the unicellular red alga Porphyridium
Source: PeerJ. 2015 Jun 18;3:e1017. doi: 10.7717/peerj.1017 (PMC4476101; doi:10.7717/peerj.1017)

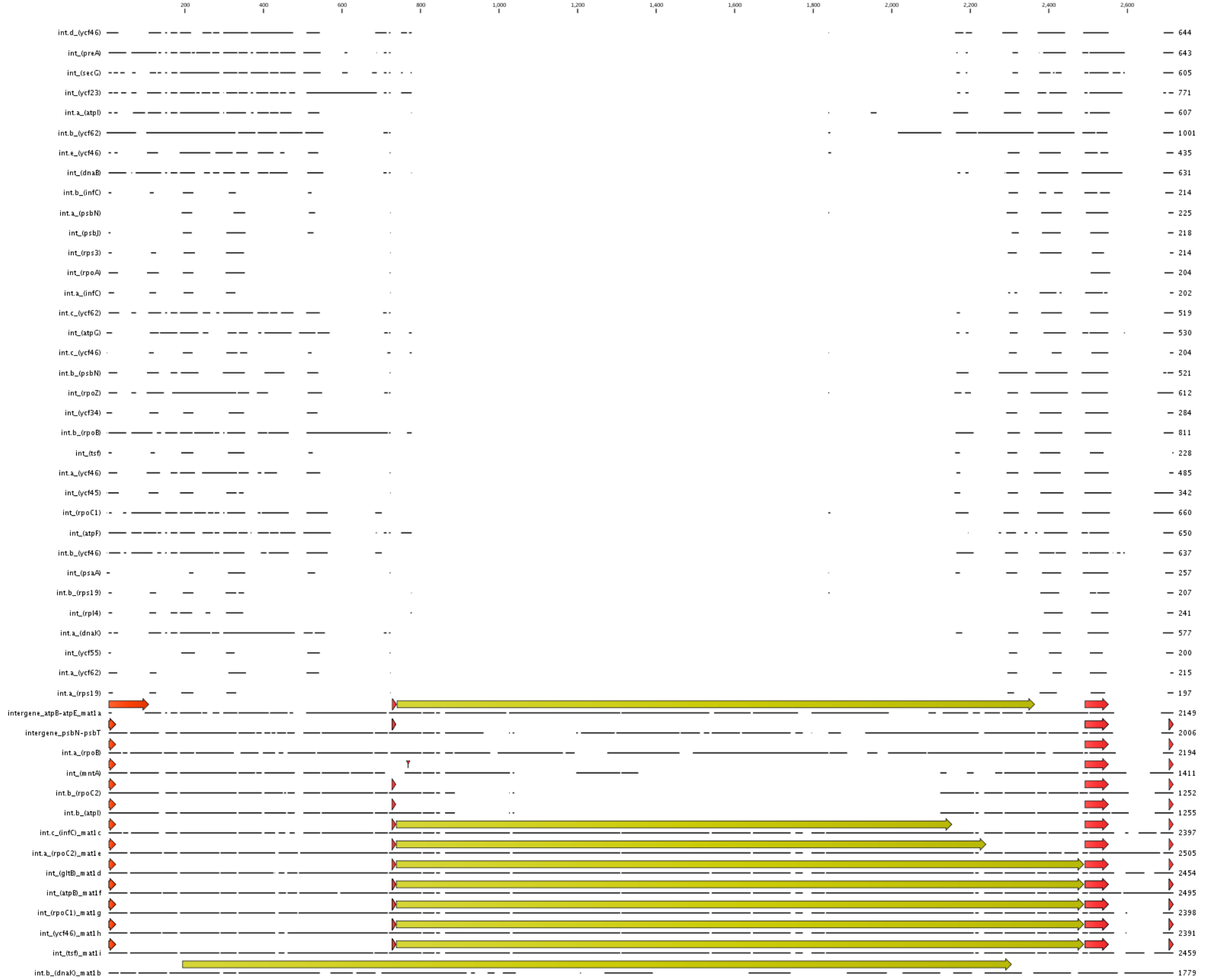

Supplement: Figure S1 — Boundaries used to determine homology are indicated in red (DI stem, DIV stem, DV and DVI stem, respectively). The IEP coding sequences are in yellow. Additional group II introns with degenerate IEPs (i.e., psbN-psbT, int.a rpoB, int mntA, int.b rpoC2) added to analysis are included. The mat1f-encoding group II intron illustrated here represents mat1fc; the nearly identical mat1fa and mat1fb are omitted. [file peerj-03-1017-s001.pdf]

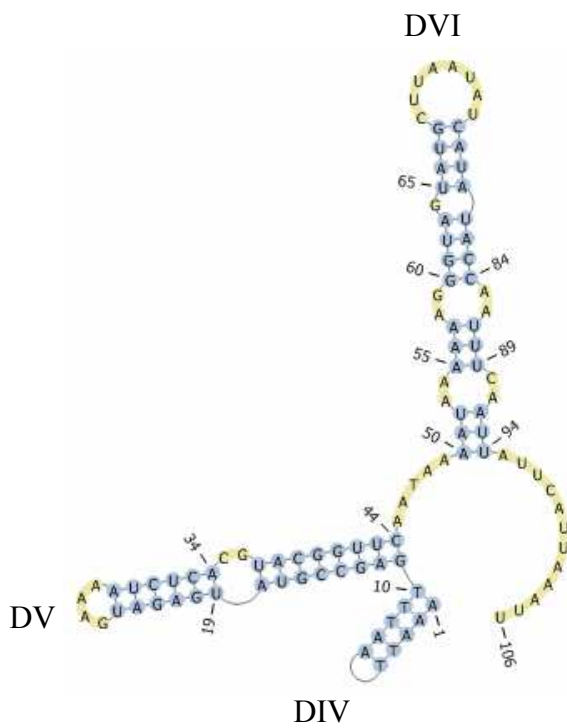

Supplement: Figure S2 — Only DIV, DV, and DVI were identified. [file peerj-03-1017-s002.pdf]

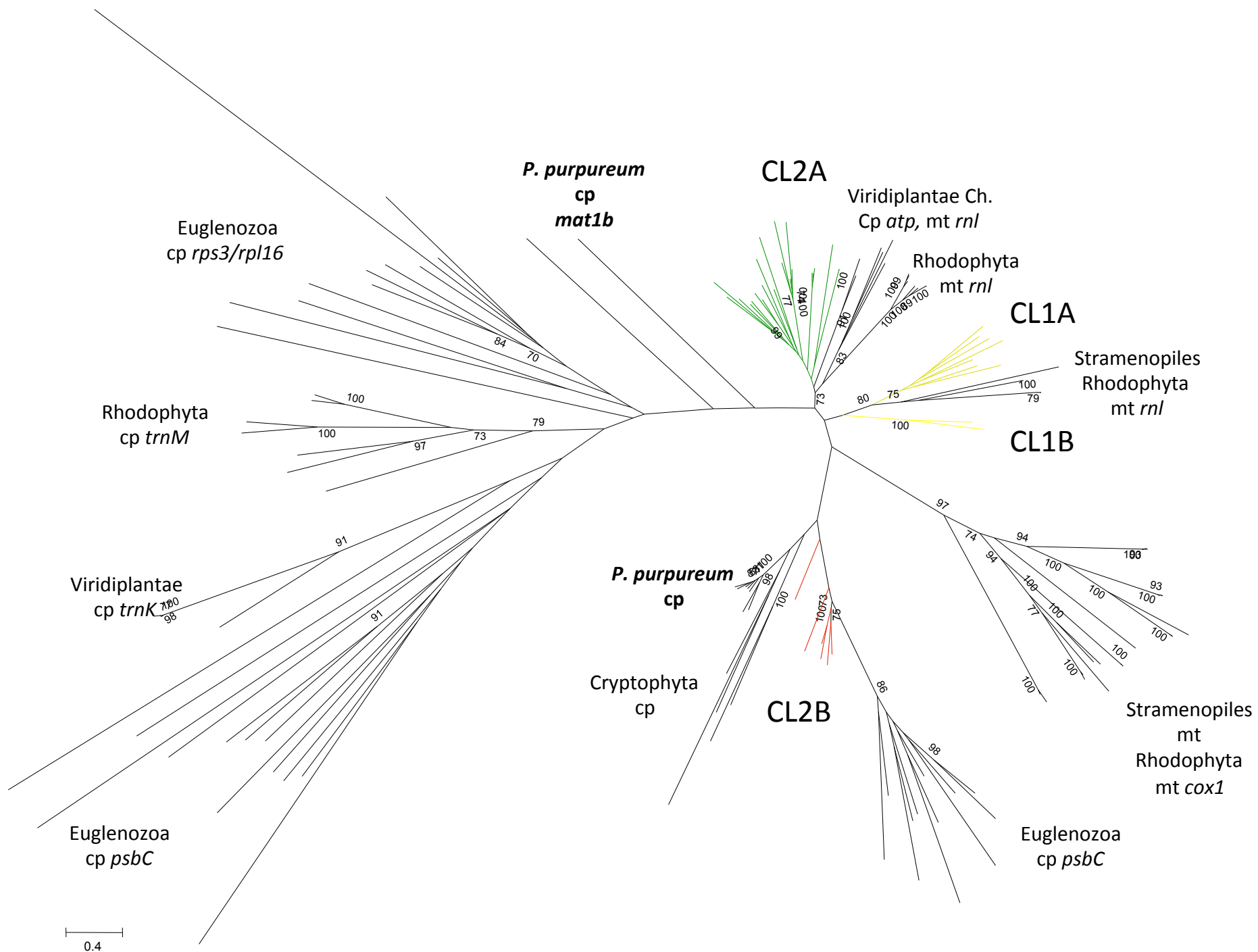

Supplement: Figure S4 — The nine plastidial IEP sequences from P. purpureum were added to selected sequences from the bacterial group II intron database, together with different eukaryote taxa such as Rhodophyta, Cryptophyta, Viridiplantae, Euglenozoa, and stramenopiles from the CL1 and CL2 group. The unrooted tree is annotated with the IEP classes (ML, bootstrap >70%). [file peerj-03-1017-s004.pdf]

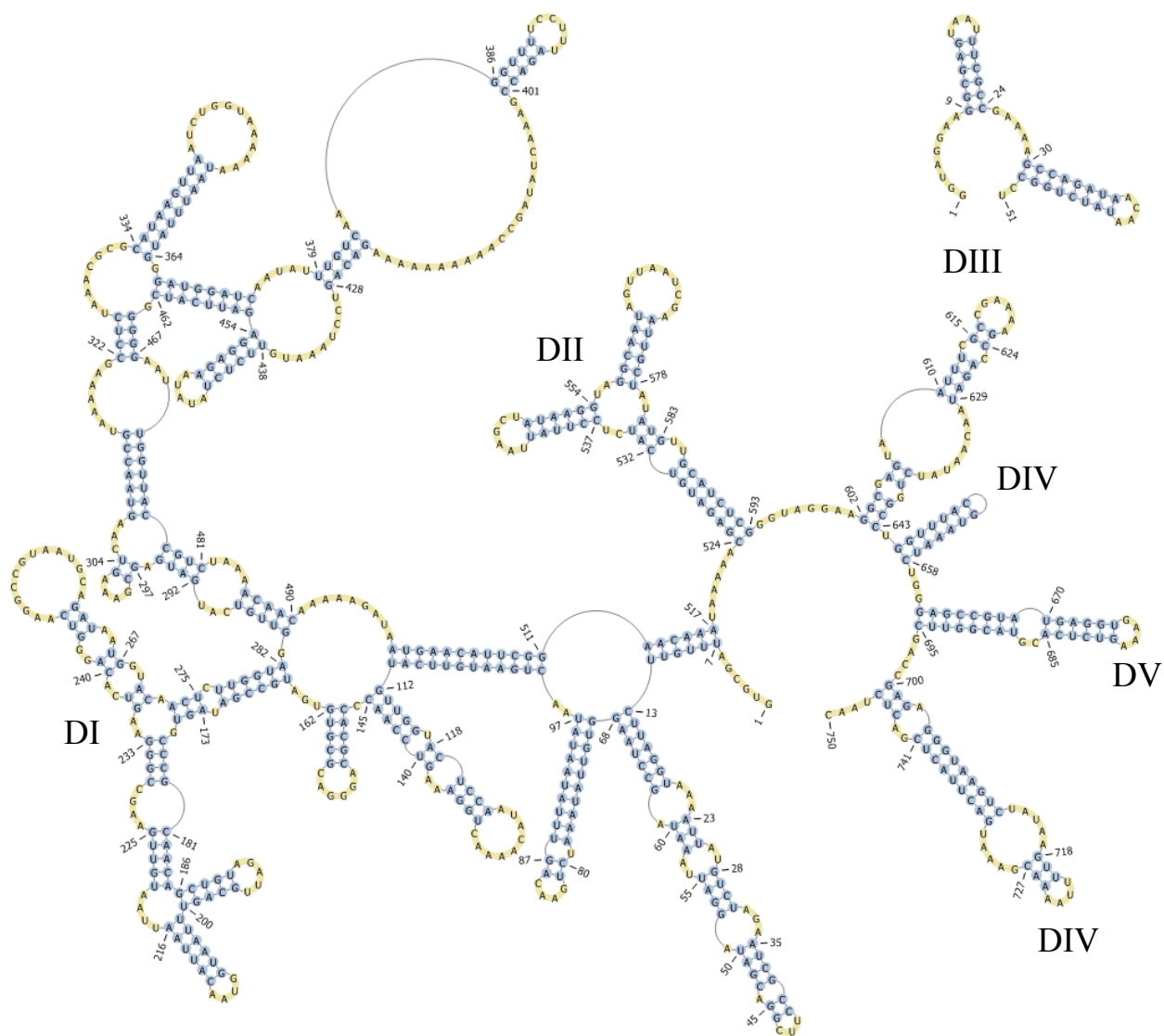

Supplement: Figure S5 — The alternate secondary structure for domain III is depicted in the floating inset. [file peerj-03-1017-s005.pdf]

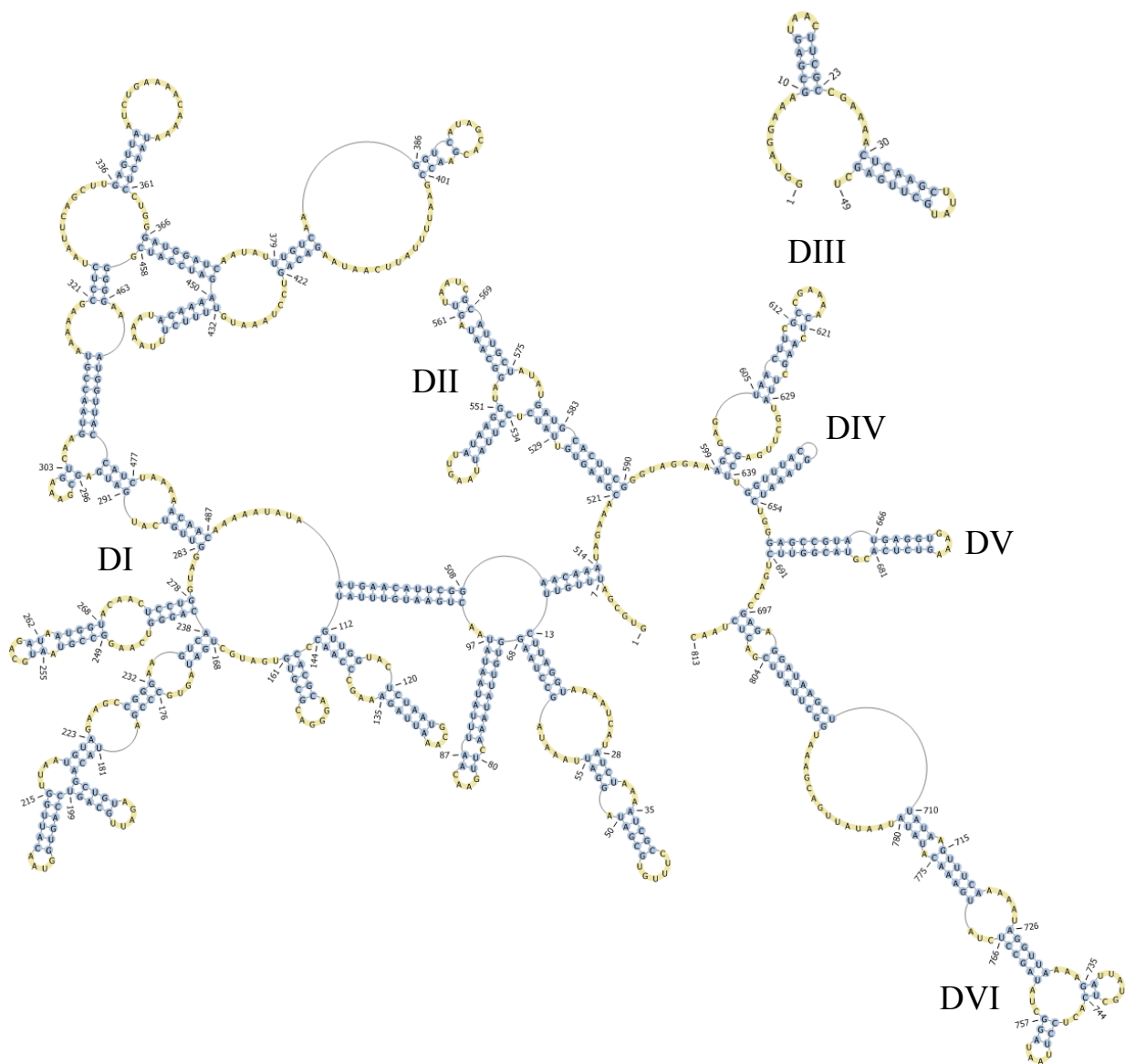

Supplement: Figure S6 — The alternate secondary structure for domain III is depicted in the floating inset. [file peerj-03-1017-s006.pdf]

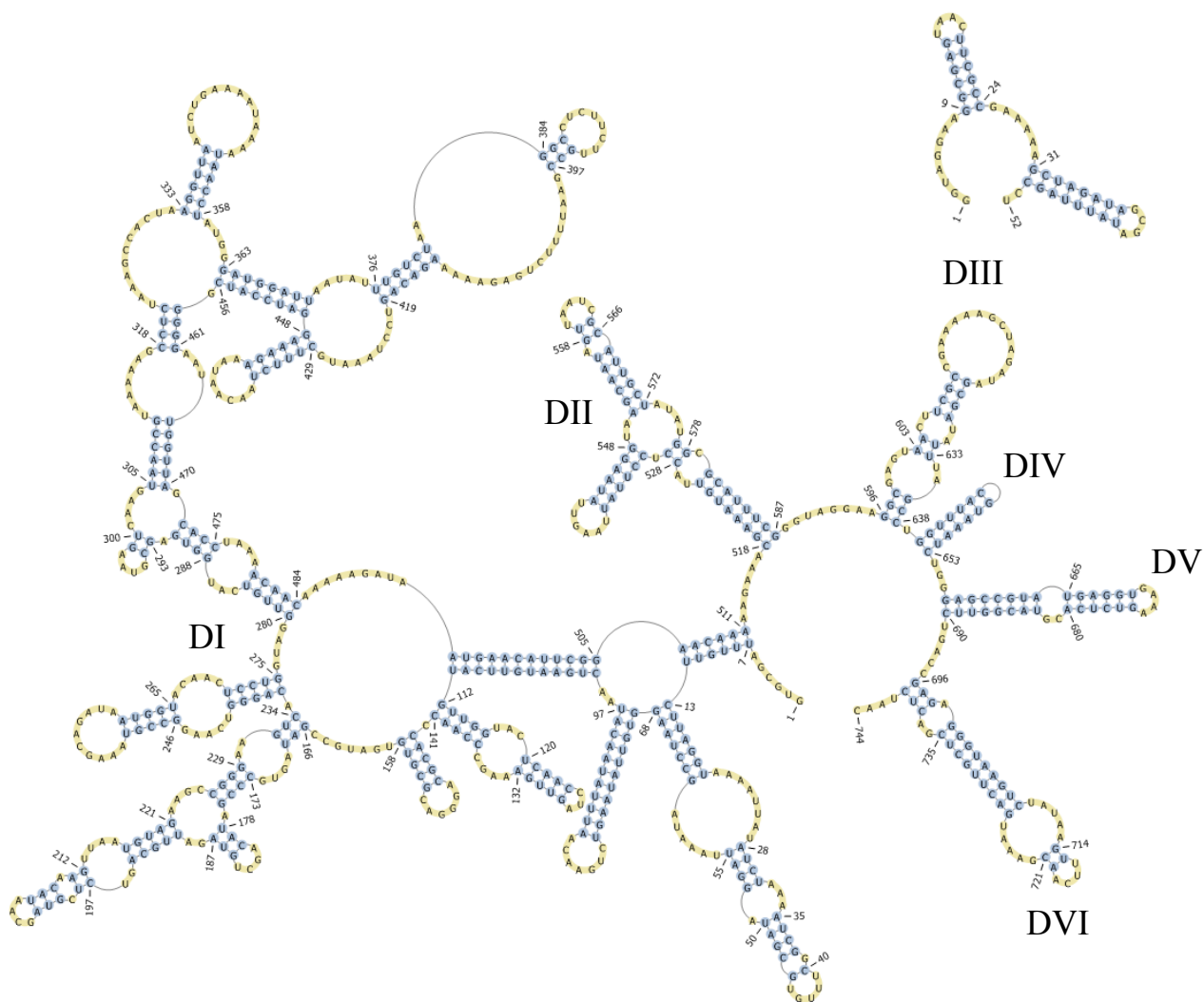

Supplement: Figure S7 — The alternate secondary structure for domain III is depicted in the floating inset. [file peerj-03-1017-s007.pdf]

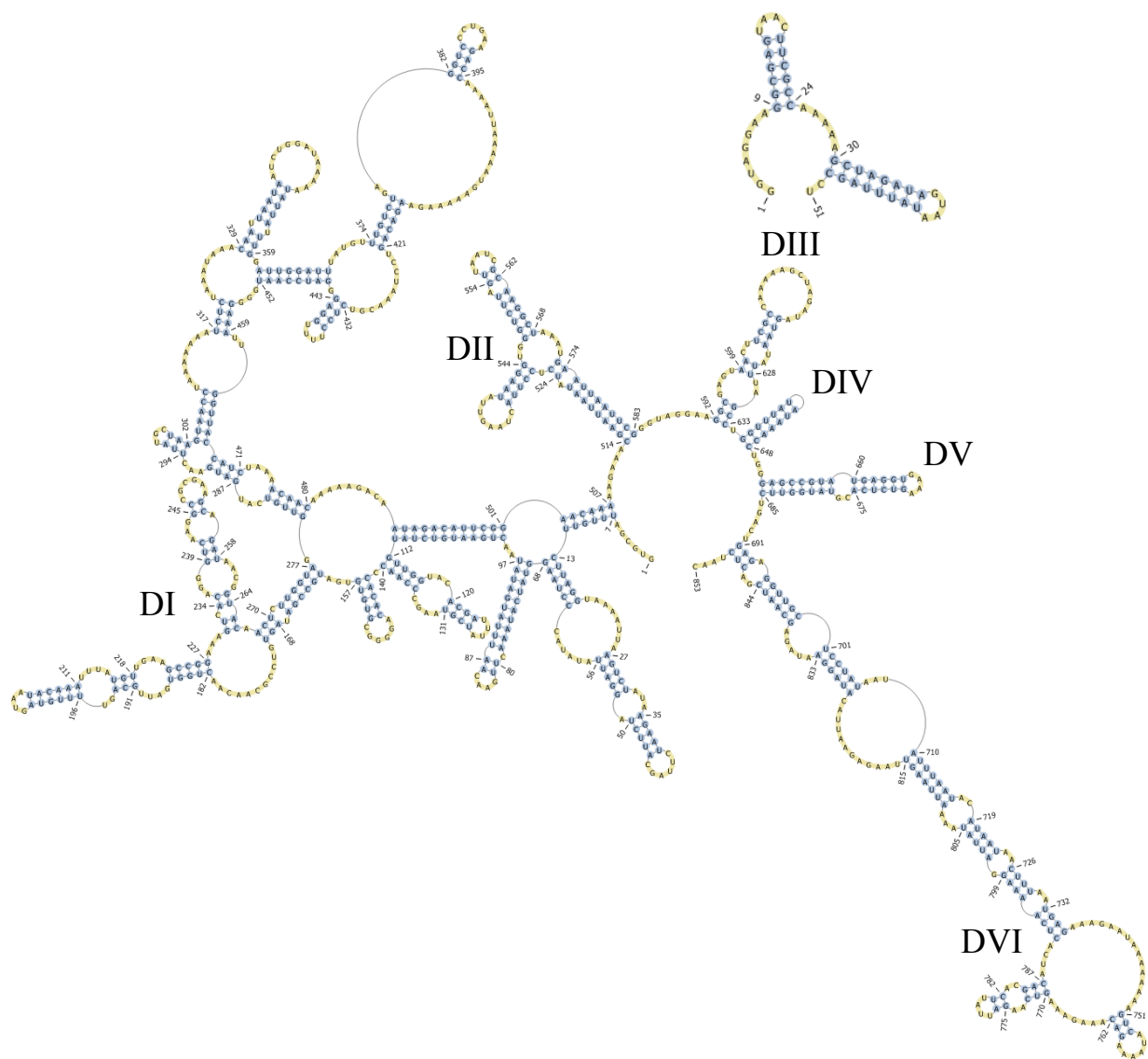

Supplement: Figure S8 — The alternate secondary structure for domain III is depicted in the floating inset. [file peerj-03-1017-s008.pdf]

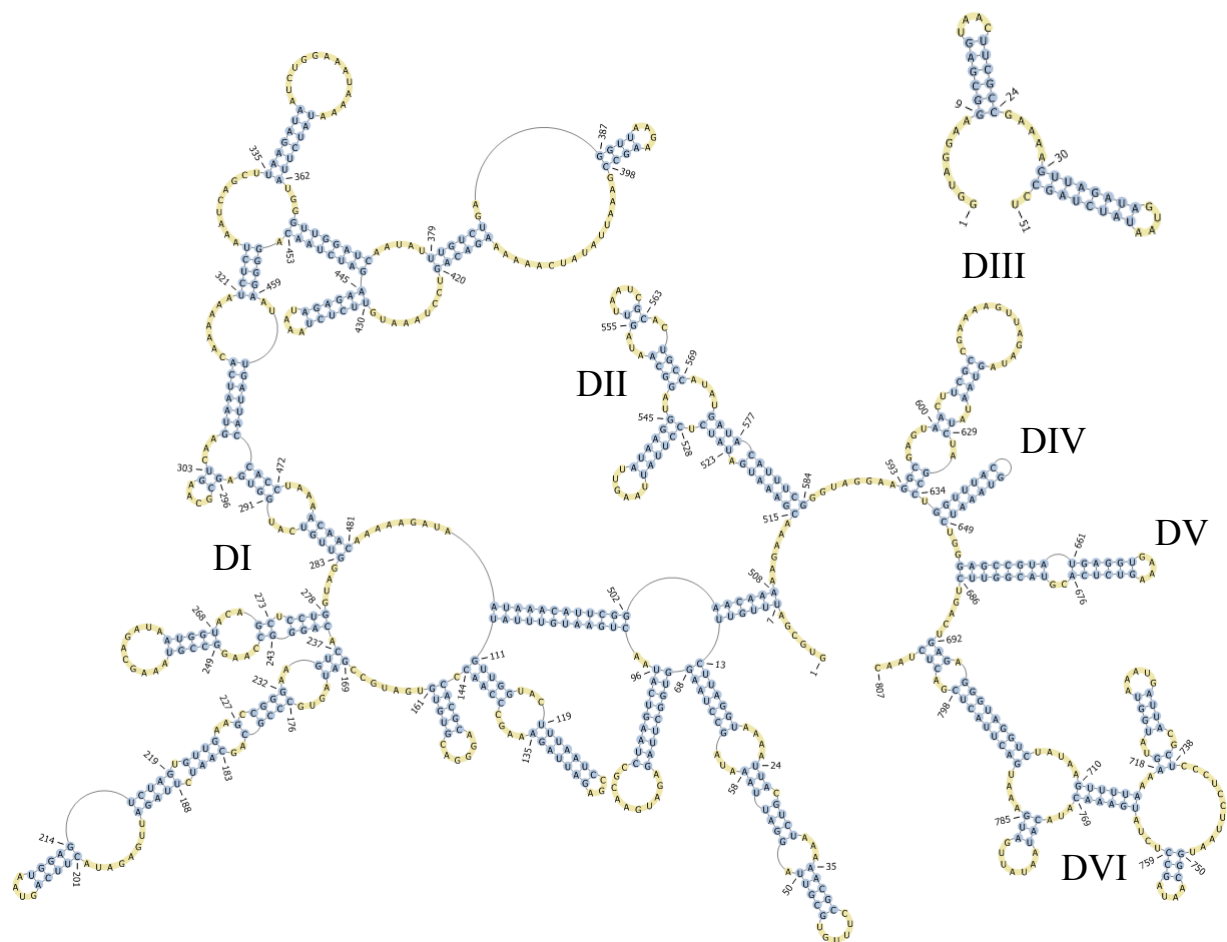

Supplement: Figure S9 — The alternate secondary structure for domain III is depicted in the floating inset. [file peerj-03-1017-s009.pdf]

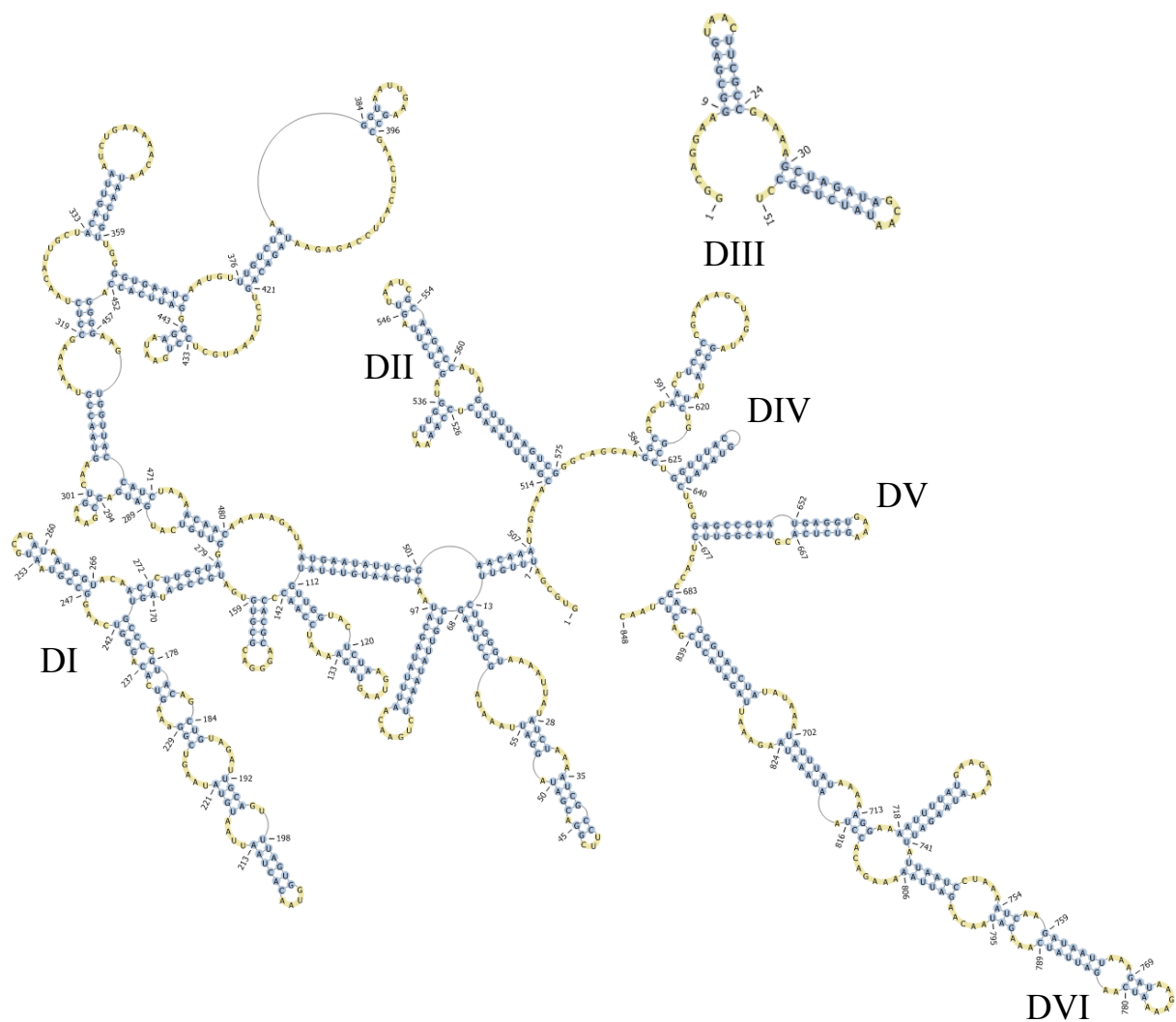

Supplement: Figure S10 — The alternate secondary structure for domain III is depicted in the floating inset. [file peerj-03-1017-s010.pdf]

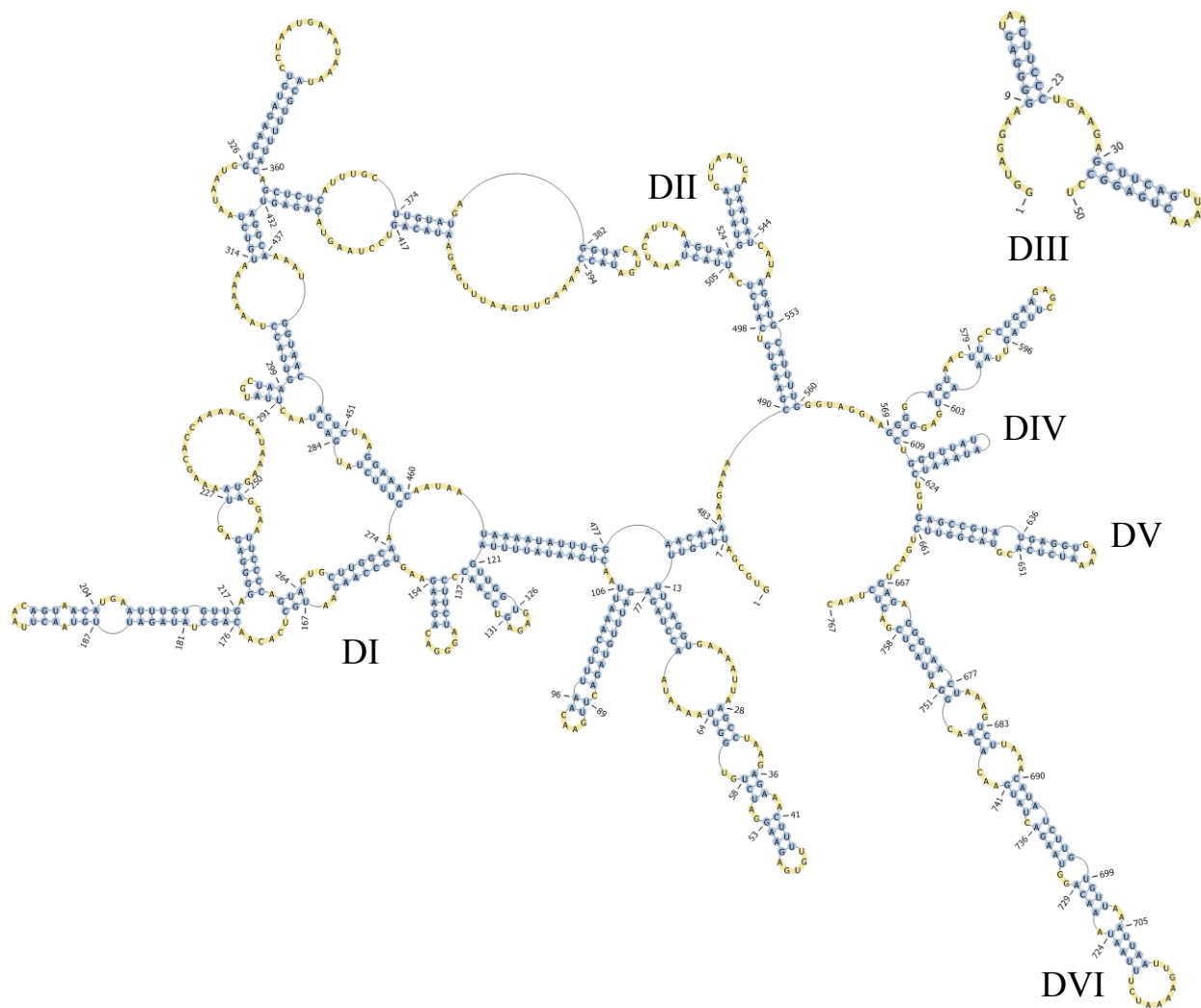

Supplement: Figure S11 — The alternate secondary structure for domain III is depicted in the floating inset. [file peerj-03-1017-s011.pdf]

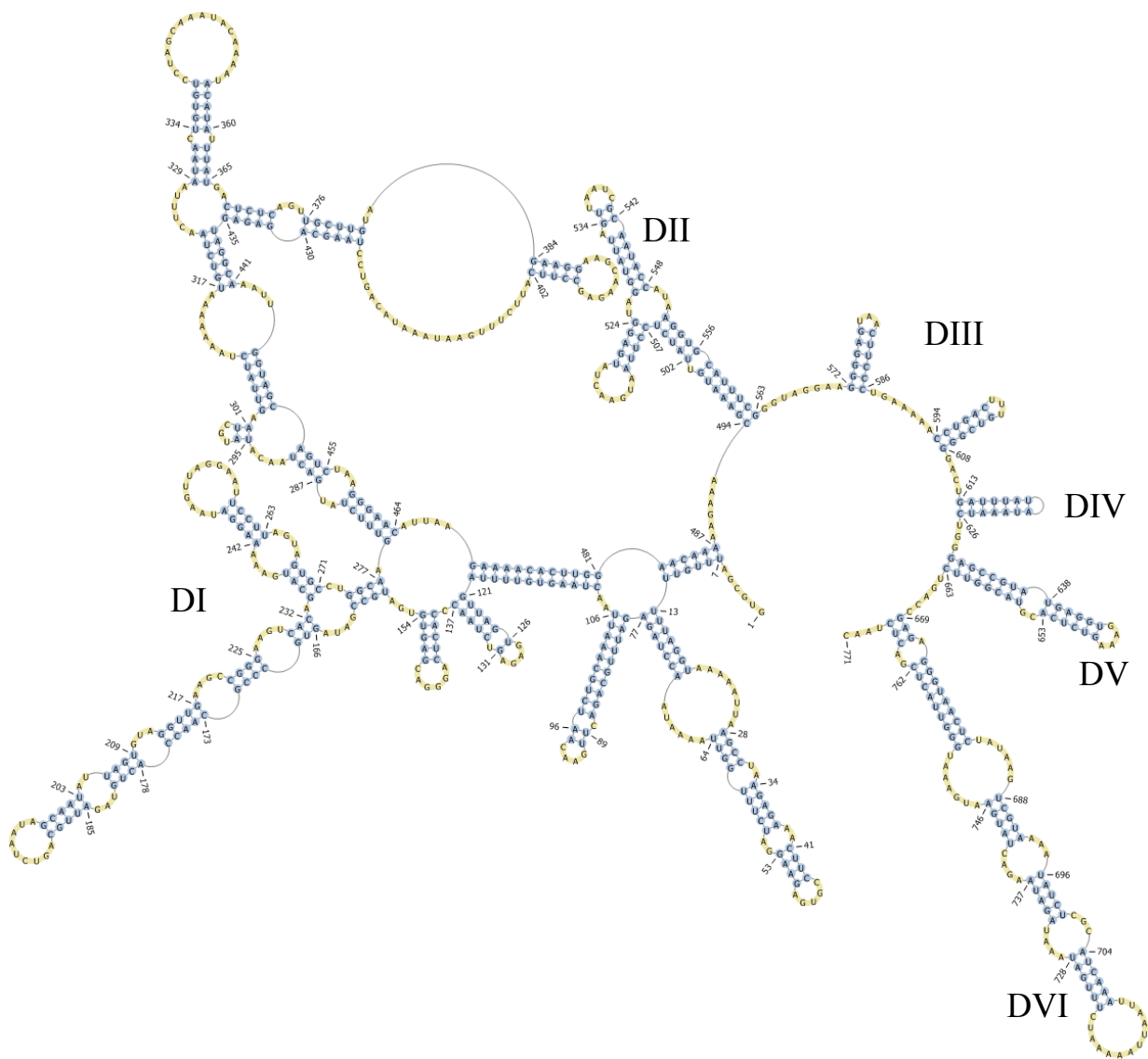

Supplement: Figure S12 [file peerj-03-1017-s012.pdf]

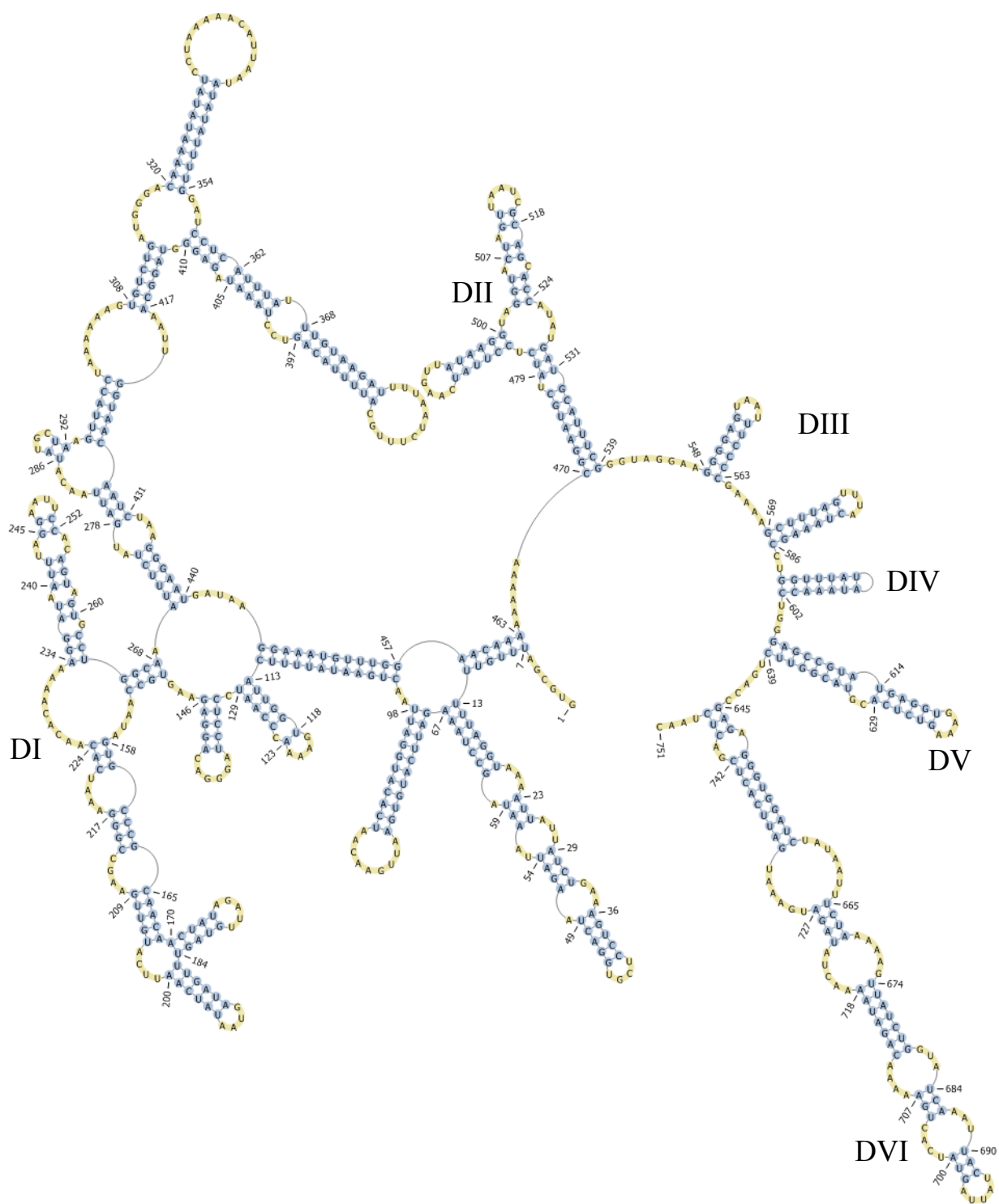

Supplement: Figure S13 [file peerj-03-1017-s013.pdf]

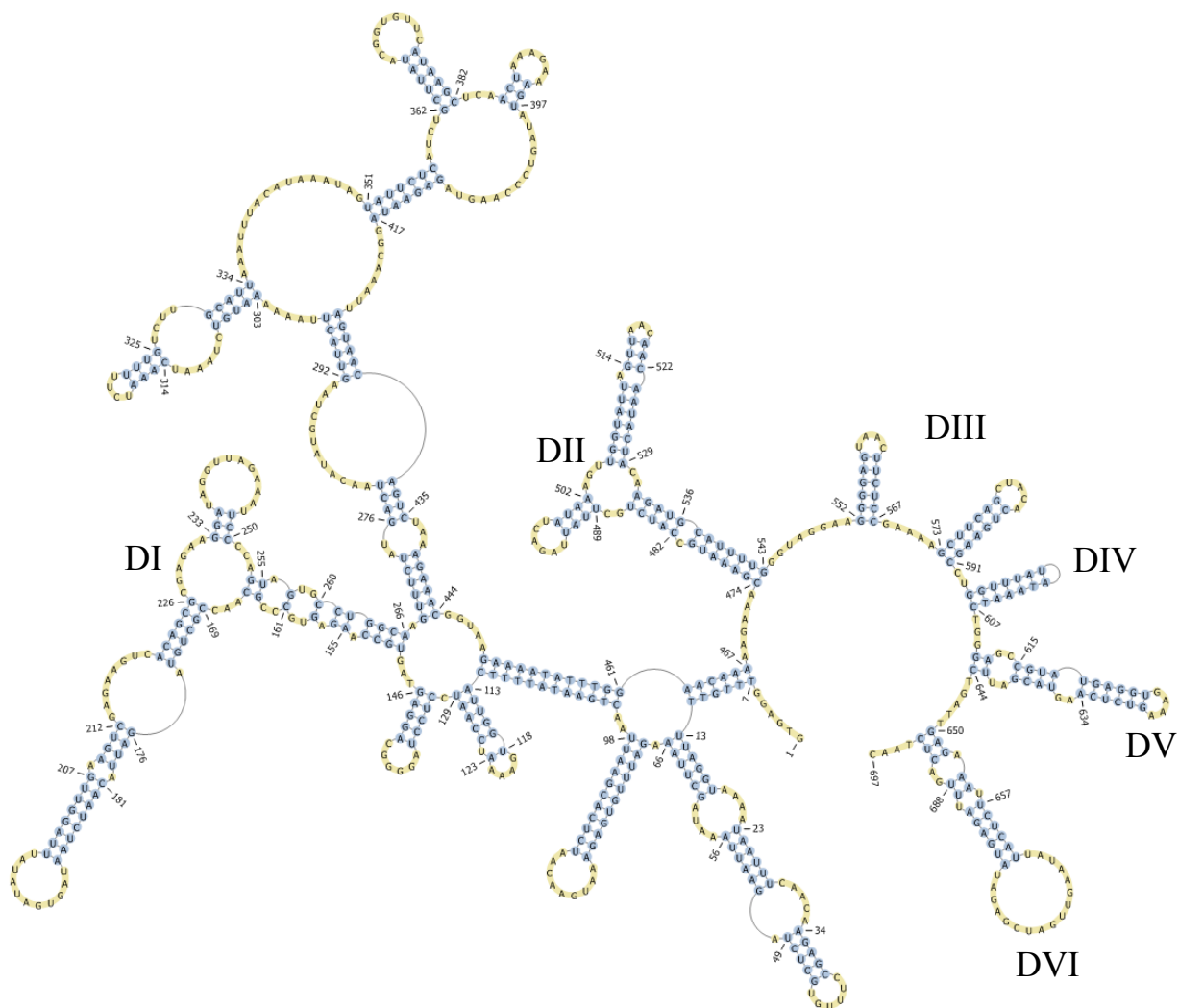

Supplement: Figure S14 [file peerj-03-1017-s014.pdf]

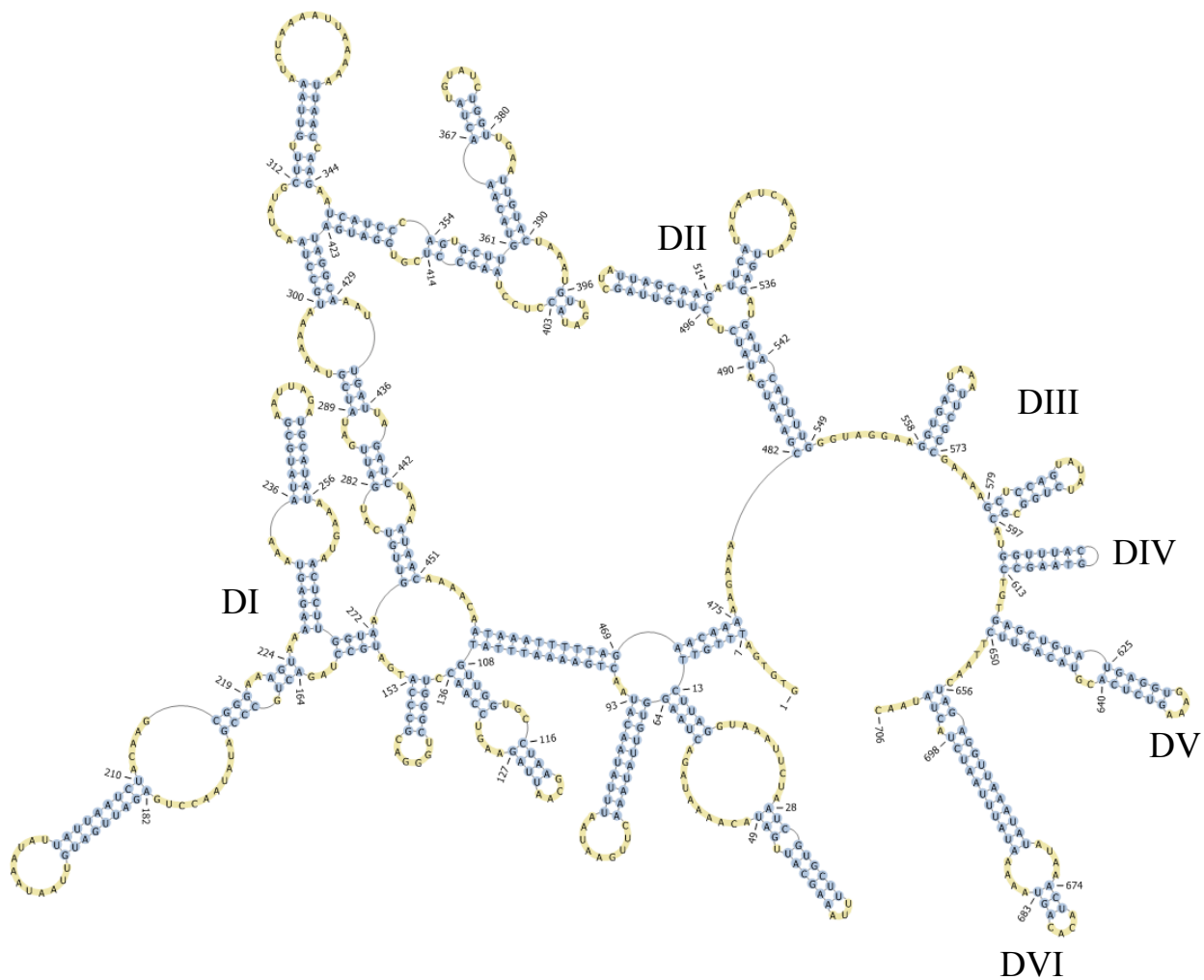

Supplement: Figure S15 [file peerj-03-1017-s015.pdf]

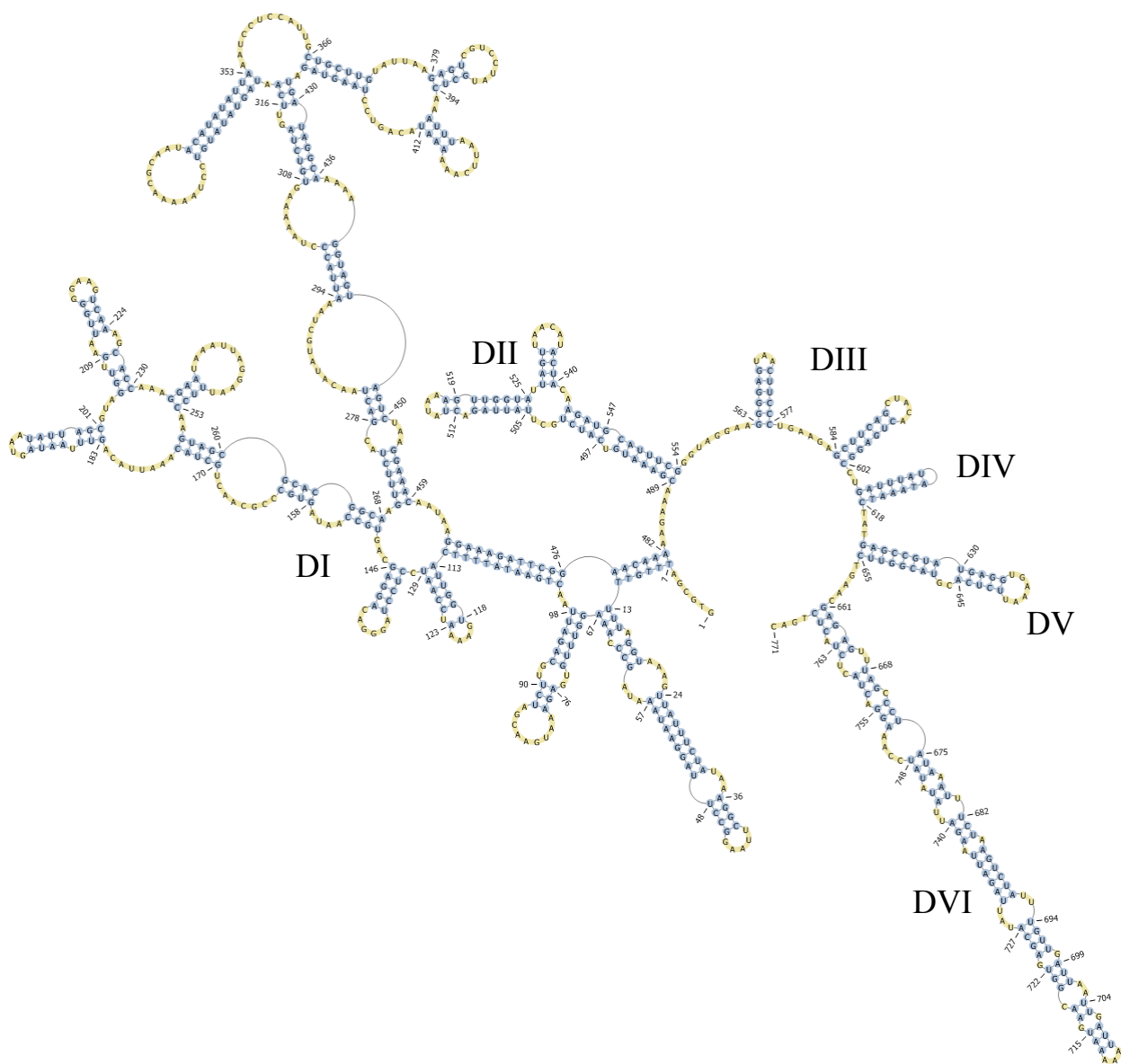

Supplement: Figure S16 [file peerj-03-1017-s016.pdf]

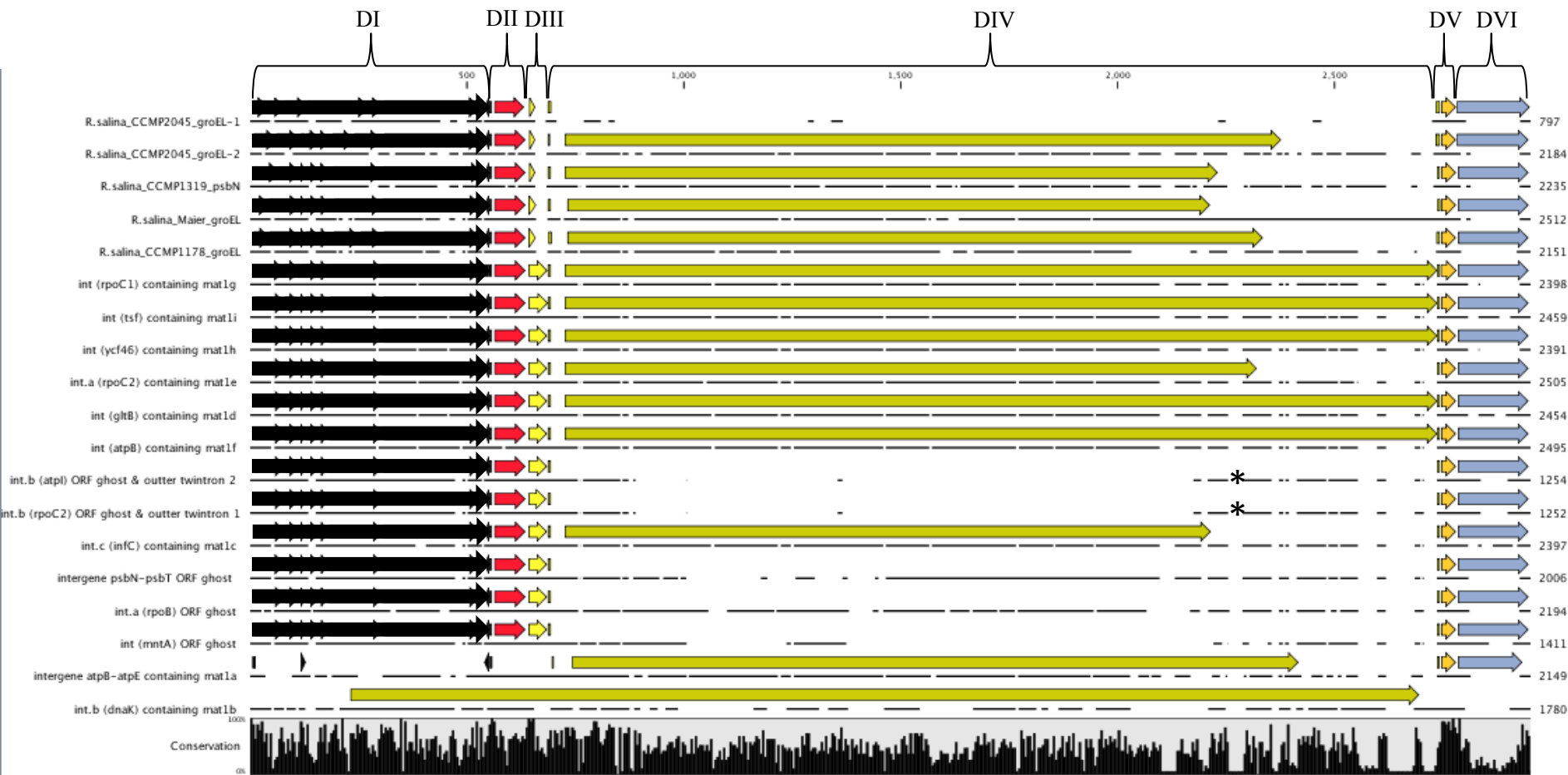

Supplement: Figure S17 — Alignment of 14 P. purpureum intron/intergenic regions containing an IEP/IEP remnant and four Rhodomonas salina introns. Secondary structures from each domain (DI–DVI) are marked and represented by different colors. The dnaK intron (containing mat1b) does not retain a group IIB intron structure. A partial structure was determined for the atpB-atpEintergenic region (containing mat1a). All the IEPs or IEP remnants are located in domain IV, including the R. salina introns (previously described as the only case of group II intron IEPs located outside of DIV). Twintron insertion sites are indicated with asterisks. The mat1f-encoding structure illustrated here is that encoding mat1fc (int.atpB); the nearly identical mat1fa- and mat1fb-encoding group II introns are omitted. [file peerj-03-1017-s017.pdf]

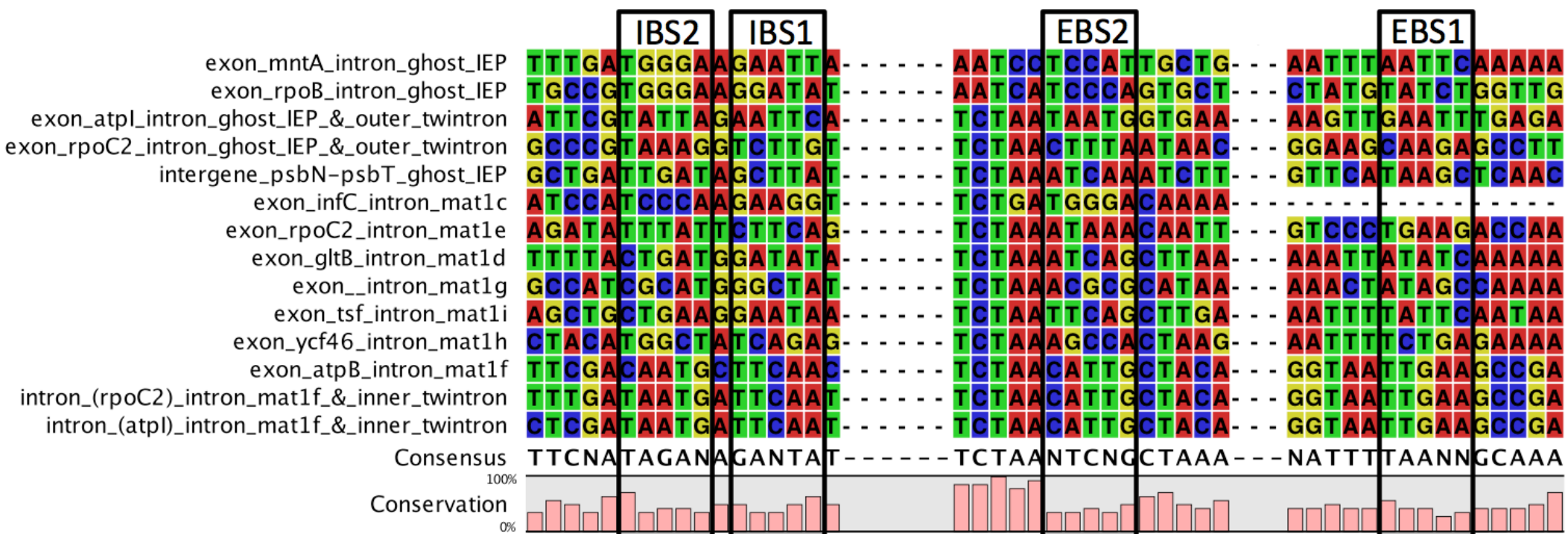

Supplement: Figure S18 — The P. purpureum EBS and IBS pairings are unique to each intron/IEP. The complementarity between both is generally preserved; if not, the mutation is located in the 5′ region. EBS1 and/or EBS2 were not identified for the mat1a, mat1b, and mat1c introns. “Ghost” refers to remnant IEPs. [file peerj-03-1017-s018.pdf]

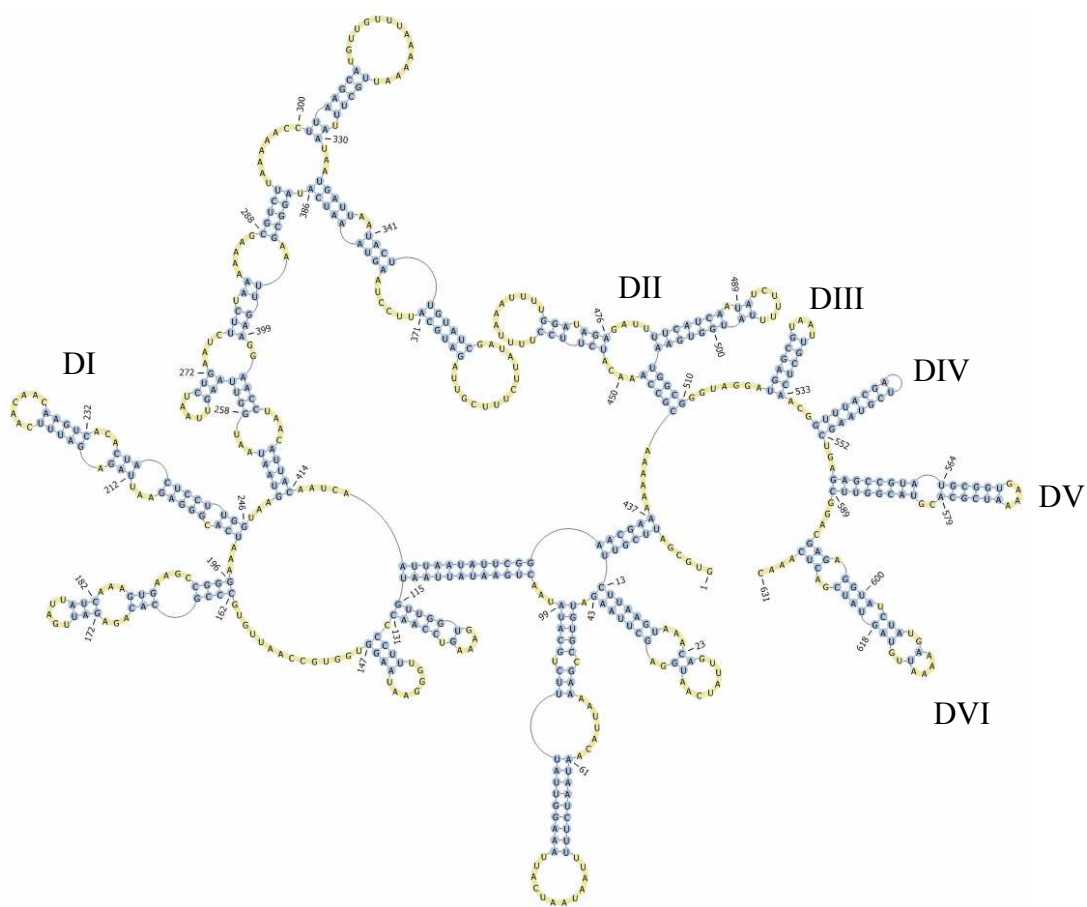

Supplement: Figure S19 — The domains II, III and IV were modified on the original structure designed by Khan et al. (2007). [file peerj-03-1017-s019.pdf]

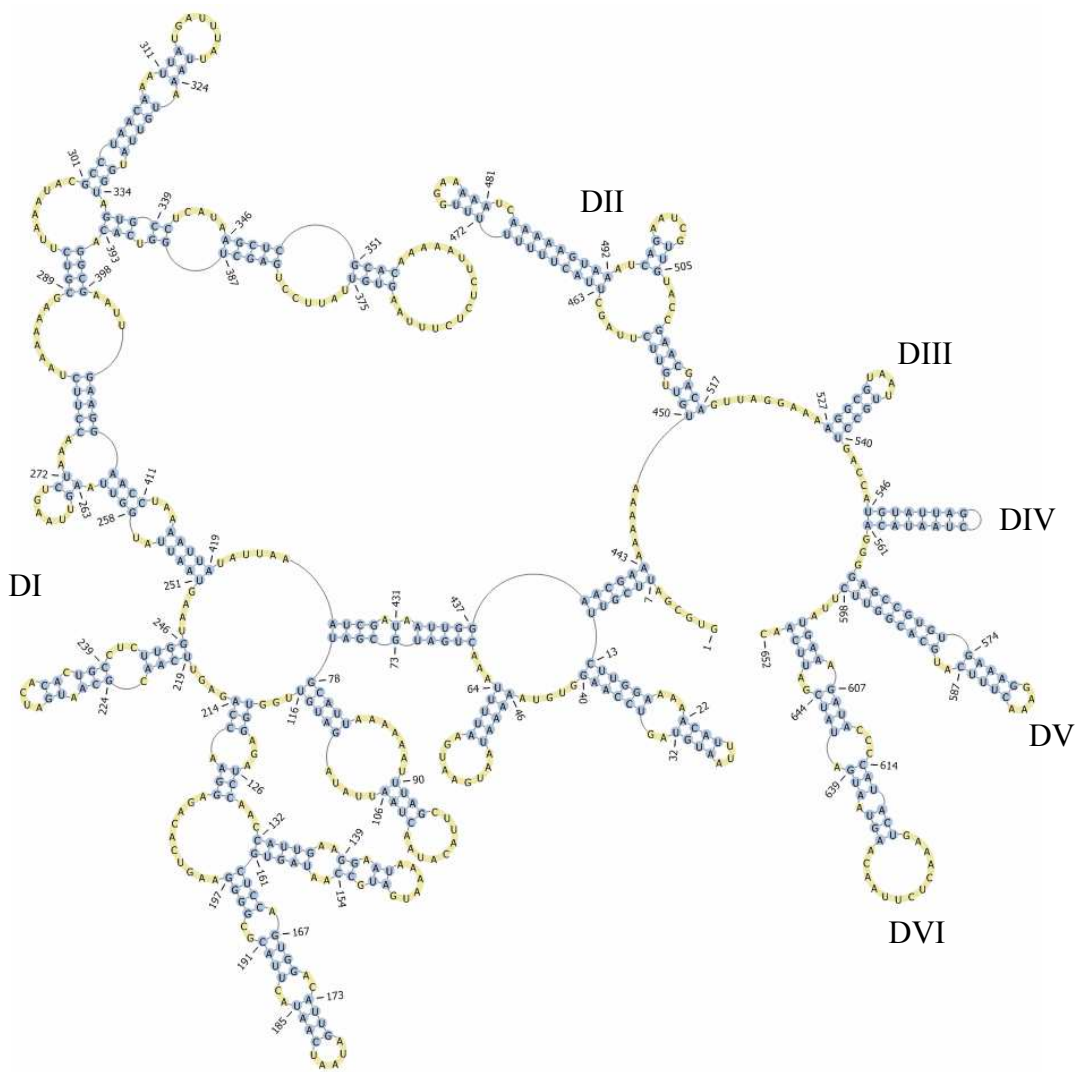

Supplement: Figure S20 — The domains II, III and IV were modified on the original structure designed by Khan et al. (2007). [file peerj-03-1017-s020.pdf]

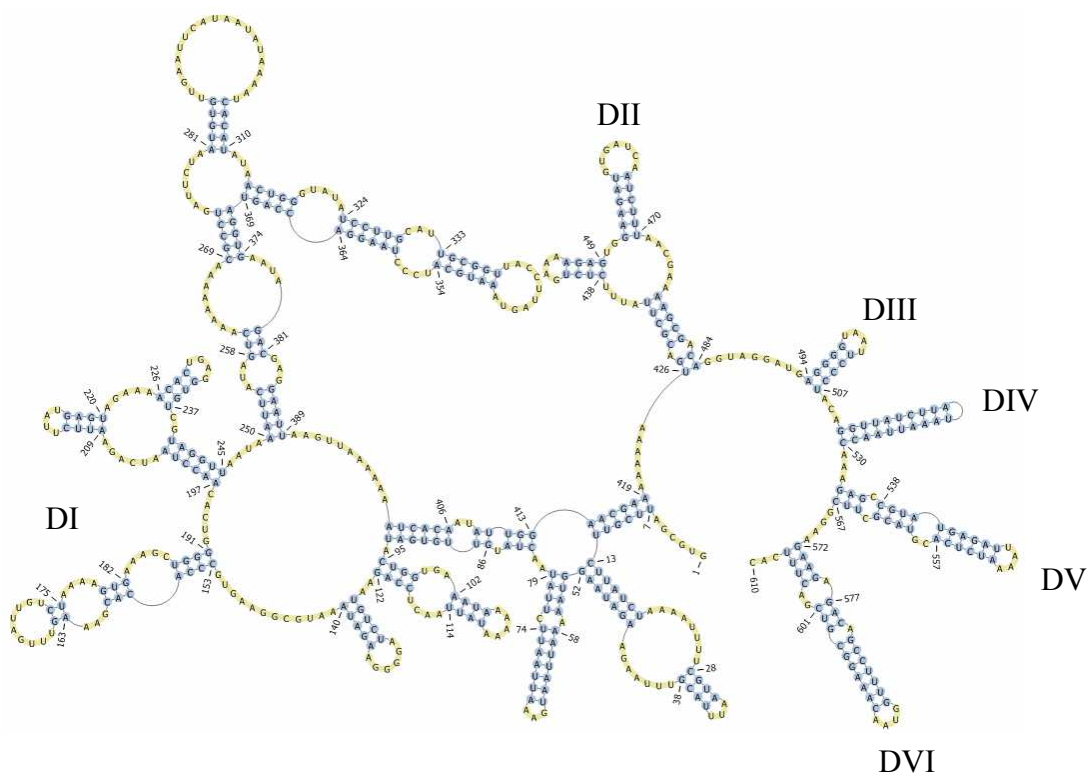

Supplement: Figure S21 — The domains III and IV were modified on the original structure designed by Khan et al. (2007). [file peerj-03-1017-s021.pdf]

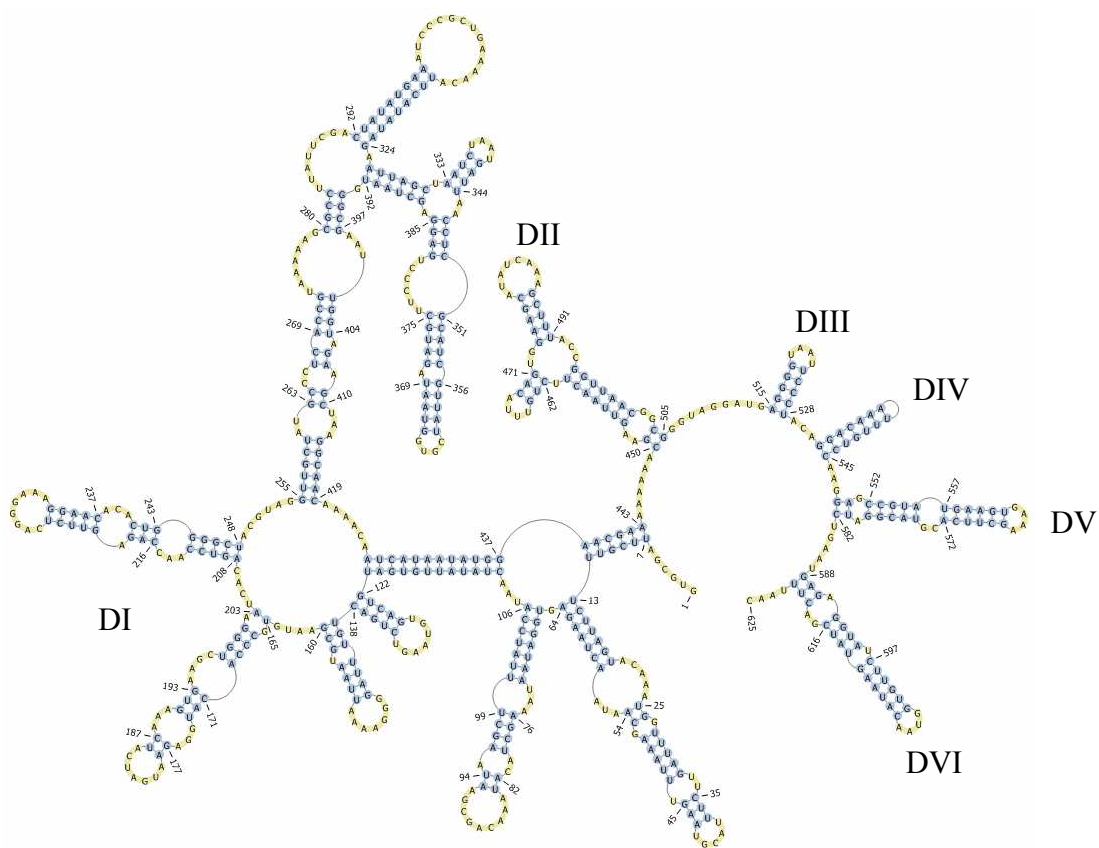

Supplement: Figure S22 — The domains I, II, III, IV and VI were modified on the original structure designed by Maier et al. (1995). [file peerj-03-1017-s022.pdf]

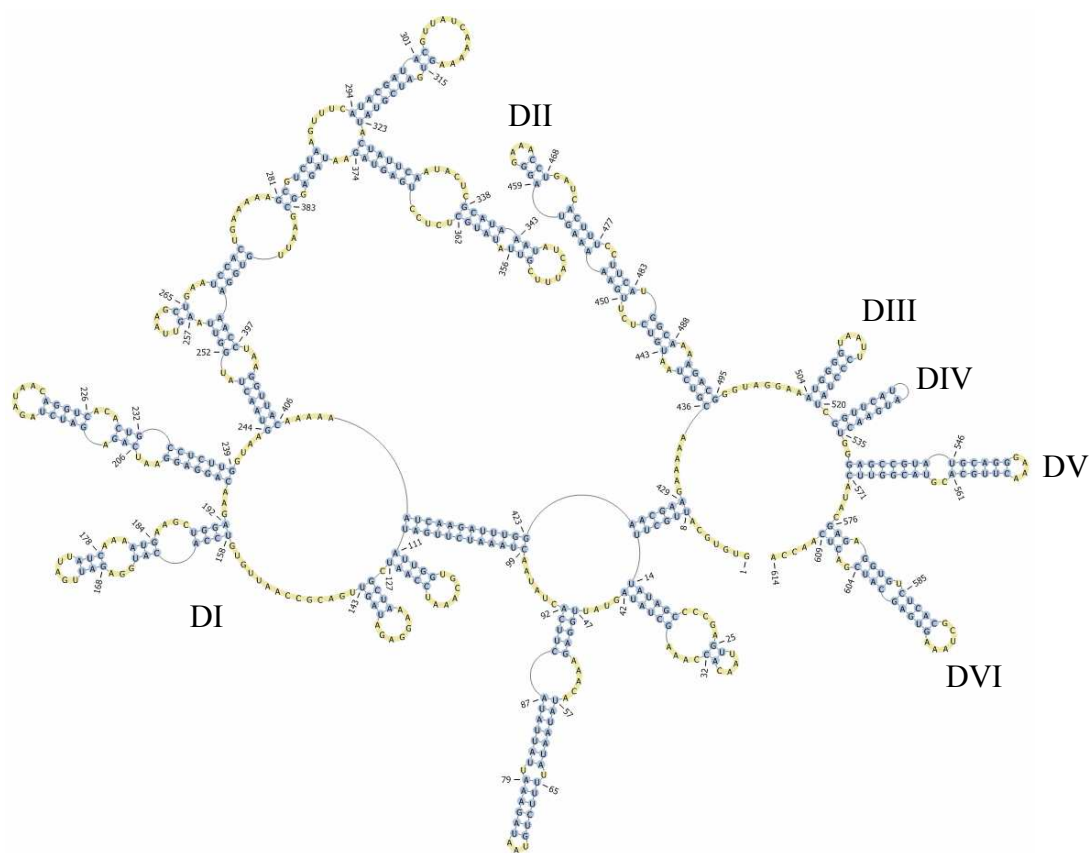

Supplement: Figure S23 — The domains I, II, III, IV and VI were modified on the original structure designed by Maier et al. (1995). [file peerj-03-1017-s023.pdf]
