# Supplementary material for: Recent mobility of plastid encoded group II introns and twintrons in five strains of the unicellular red alga Porphyridium
Source: PeerJ. 2015 Jun 18;3:e1017. doi: 10.7717/peerj.1017 (PMC4476101; doi:10.7717/peerj.1017)
Supplement: Figure S3 — The four identified domains are separated by an artificial five amino acid gap. The unboxed 5′ sequence comprises the reverse transcriptase (RT) domain. The maturase (X) domain is boxed in black, the DNA-binding (D) domain in red and the endonuclease (En) domain in blue. The D and En domains are partial or absent in four IEPs (mat1a, mat1b, mat1c and mat1e). Asterisks are placed above the YADD domain. [file peerj-03-1017-s003.pdf]

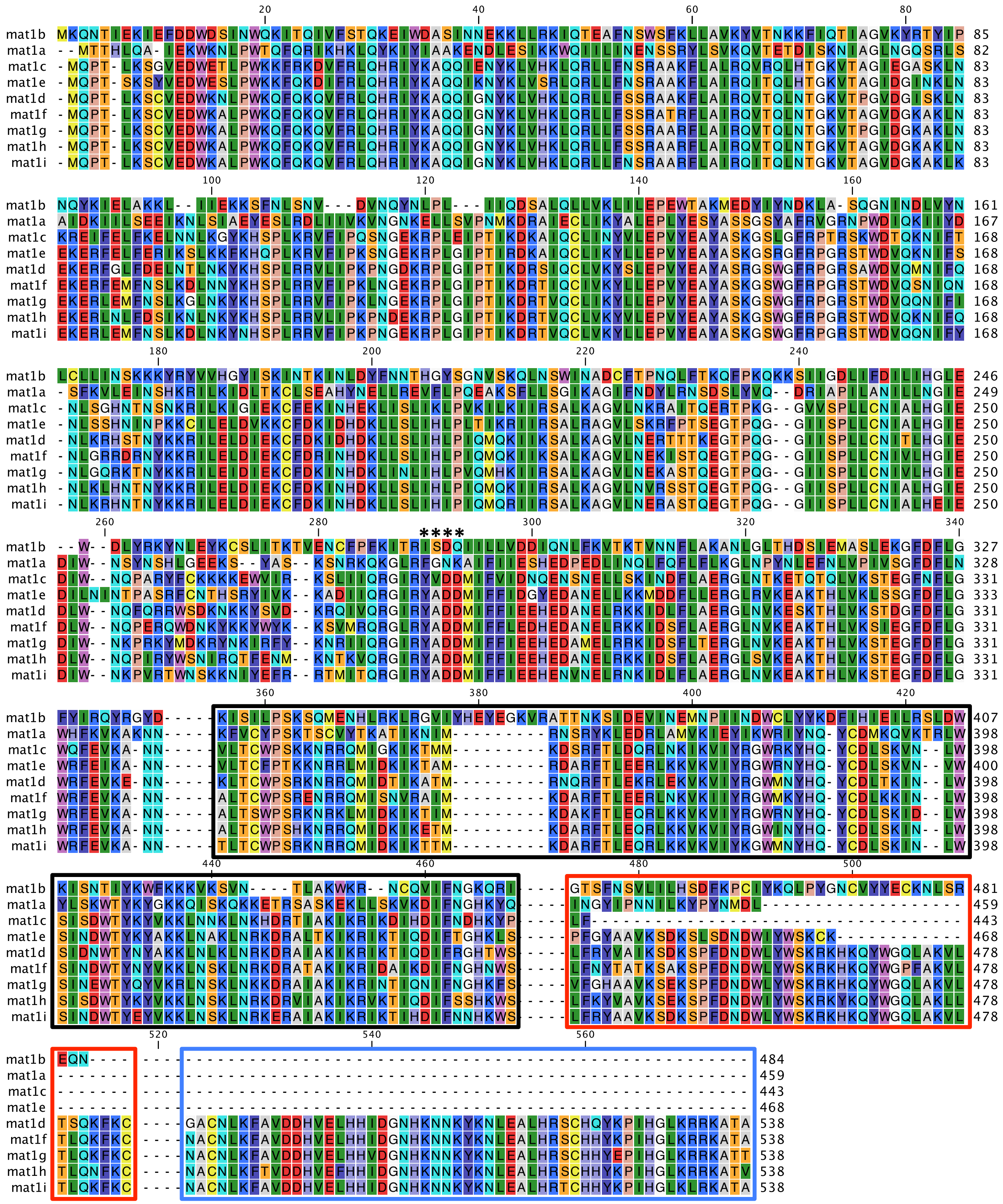
mat1b
mat1a
mat1c
mat1e
mat1d
mat1f
mat1g
mat1h
mat1i
20
40
60
80
100
120
140
160
180
200
220
240
260
280
300
320
340
360
380
400
420
440
460
480
500
520
540
560
****
