# Supplementary material for: Recent mobility of plastid encoded group II introns and twintrons in five strains of the unicellular red alga Porphyridium
Source: PeerJ. 2015 Jun 18;3:e1017. doi: 10.7717/peerj.1017 (PMC4476101; doi:10.7717/peerj.1017)
Supplement: Figure S24 — The binding sites of the maturases were determined by comparing sequence alignments. The stem-loop structure from a purine-rich internal loop is framed in white, whereas the start- codon is framed in black. [file peerj-03-1017-s024.pdf]

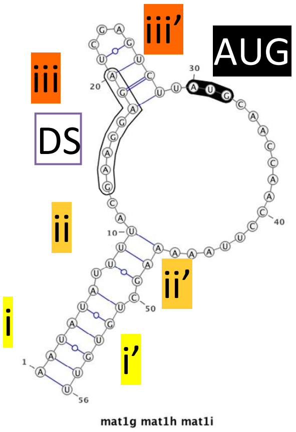
iii'
AUG
iii
DS
ii
ii'
i
i'
mat1g mat1h mat1i

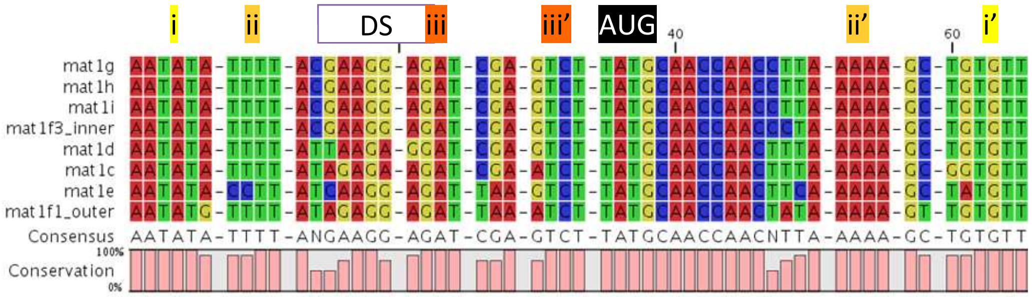
i
ii
DS
iii
iii'
AUG
40
ii'
60
i'
mat1g AATATA-TTTT-ACGAAGG-AGAT-CGA-GTCT-TATGCAACCAACCTTA-AAAA-GC-TGTGTT
mat1h AATATA-TTTT-ACGAAGG-AGAT-CGA-GTCT-TATGCAACCAACCTTA-AAAA-GC-TGTGTT
mat1i AATATA-TTTT-ACGAAGG-AGAT-CGA-GTCT-TATGCAACCAACCTTA-AAAA-GC-TGTGTT
mat1f3_inner AATATA-TTTT-ACGAAGG-AGAT-CGA-GTCT-TATGCAACCAACCCTA-AAAA-GC-TGTGTT
mat1d AATATA-TTTT-ATTAAGA-GGAT-CGA-GTCT-TATGCAACCAACTTTA-AAAA-GC-TGTGTT
mat1c AATATA-TTTT-ATAGAGA-AGAT-CGA-ATCT-TATGCAACCAACTTTA-AAAA-GC-GGTGTT
mat1e AATATA-CCTT-ATCAAGG-AGAT-TAA-GTCT-TATGCAACCAACTTCA-AAAA-GC-TATGTT
mat1f1_outer AATATG-TTTT-ATAGAGG-AGAT-TAA-ATCT-TATGCAACCAACTATA-AAAA-GT-TGTGTT
Consensus AATATA-TTTT-ANGAAGG-AGAT-CGA-GTCT-TATGCAACCAACNTTA-AAAA-GC-TGTGTT
100%
Conservation
0%
